# Characterization of the complete chloroplast genome of a Peruvian landrace of *Capsicum chinense* Jacq. (Solanaceae), arnaucho chili pepper

**DOI:** 10.1080/23802359.2021.2014366

**Published:** 2022-01-05

**Authors:** Carlos I. Arbizu, Carla L. Saldaña, Rubén D. Ferro-Mauricio, Julio C. Chávez-Galarza, Jordan Herrera, Sergio Contreras-Liza, Juan C. Guerrero-Abad, Jorge L. Maicelo

**Affiliations:** a Dirección de Desarrollo Tecnológico Agrario, Instituto Nacional de Innovación Agraria (INIA), La Molina, Lima, Perú; b Facultad de Ingeniería Agrarias, Industrias Alimentarias y Ambiental, Universidad Nacional José Faustino Sánchez Carrión, Huacho, Lima, Perú; c Dirección de Recursos Genéticos y Biotecnología, Instituto Nacional de Innovación Agraria (INIA), La Molina, Lima, Perú

**Keywords:** NGS, chloroplast, genetic resources, arnaucho, phylogenomics

## Abstract

In this study, we sequenced the first complete chloroplast (cp) genome of a Peruvian chili pepper landrace, “arnacucho” (*Capsicum chinense*). This cp genome has a 156,931 bp in length with typical quadripartite structure, containing a large single copy (LSC) region (87,325 bp) and a 17,912 bp small single-copy (SSC) region, separated by two inverted repeat (IR) regions (25,847 bp); and the percentage of GC content was 37.71%. Arnaucho chili pepper chloroplast genome possesses 133 genes that consists of 86 protein-coding genes, 37 tRNA, eight rRNA, and two pseudogenes. Phylogenetic analysis revealed that this Peruvian chili pepper landrace is closely related to the undomesticated species *C. galapagoense*; all belong to the Capsiceae tribe.

Peru harbors the largest morphological diversity of cultivated chili peppers worldwide (Meckelmann et al. 2013), and they play a crucial role in the Peruvian cuisine and cultural traditions (Morales-Soriano et al. 2018). Arnaucho chili pepper is a landrace cultivated by small farmers in a restricted area named ‘Valle de Supe’ (around 200 km northern Lima) and is an important representative in the gastronomy of Lima region. This landrace possesses an annular constriction of the calyx, two flowers per axil, red ripe fruit color, triangular fruit shape, pointed fruit apex shape, greenish-yellow corolla (Aliaga et al. 2019). Even though NGS techniques are being employed to study *Capsicum* L. spp. genomes, very little is known about the genomics of Peruvian chili pepper. In addition, knowledge about Peruvian *Capsicum chinense* Jacquin 1777 phylogenetic relationships is scarce. Therefore, we here report the first complete chloroplast genome (cp) of a Peruvian chili pepper landrace by next-generation sequencing technology. Moreover, a phylogenetic tree of this species and its relatives is presented.

We collected young fresh leaves of arnaucho chili pepper from the Lima region (−10.8099889, −77.6953950). This specimen was deposited at the Germplasm Bank of INIA (https://www.gob.pe/inia, drgb@inia.gob.pe) under the voucher number PER1002642. Total genomic DNA was extracted by CTAB method (Doyle and Doyle 1990). Pair-end clean reads were obtained by PE 150 library and the Illumina HiSeq 2500 platform. Adapters and low-quality reads were removed using Trim Galore (Martin 2011). We assembled the chloroplast genome using the GetOrganelle v1.7.2 pipeline (Jin et al 2020). Chloroplast genome was annotated with GeSeq in CHLOROBOX web service (Tillich et al 2017).

The total length of the chloroplast genome is 156,931 bp, which is 1,635 bp longer than one of the most economically important species in the Solanaceae family, potato (*Solanum tuberosum*). This cp genome presents a typical quadripartite structure, containing 87,325 bp as large single copy (LSC) region and 17,912 bp as small single-copy (SSC) region, separated by two inverted repeats (IR) regions (25,847 bp). The total GC content was 37.71%. Arnaucho chili pepper chloroplast genome contains 133 genes, including 86 protein-coding genes, 37 tRNA genes, 8 rRNA genes, and 2 pseudogenes. Similarly, chloroplast genome of the other two *C. chinense* (KX913217, KU041709) and *C. galapagoense* Hunziker 1956 (NC_033524) presents 86 protein-coding genes, 45 non-coding regions and 2 pseudogenes Most of these genes did not contain an intron; only 25 genes harbored one intron, and two genes (pafI, clpP1) contained two introns. Most genes occurred as a single copy, except 18 genes that were duplicated in IR regions. On the other hand, D’Agostino et al. (2018) indicated that the plastome size of 11 *Capsicum* genotypes ranged from 156,836 bp in *C. frutescens* Linaeus 1753 to 157,390 in *C. pubescens* Ruiz & Pavon 1799. Moreover, they mentioned those plastomes contained 113 unique genes, 79 protein-coding, 4 rRNA, and 30 tRNA genes. The chloroplast genome sequence and annotation were submitted to NCBI with accession number MZ379791.

We constructed a maximum likelihood (ML) phylogenetic tree of 17 genomes obtained from GenBank. Each genome was aligned by MAFFT v7.475 (Katoh and Standley 2013). Then, we used GTR + GAMMA model of evolution to obtain the best-scoring ML tree, and then 1,000 nonparametric bootstrap inferences were performed with RAxML v8.2.11 (Stamatakis 2014). The phylogenomic analyses were consistent with a previous study that also employed plastome sequences (D’Agostino et al. 2018; Sebastin et al. 2019). Interestingly, ML phylogenetic analysis showed that arnacucho chili pepper is sister to *C. galapagoense* and sister to them is a clade containing *C. chinense* and *C. eximium* Hunziker 1950 (Figure 1). This chili pepper landrace is considered a *C. chinense* species by many authors based mainly on morphological characters (Aliaga et al. 2019). However, its current taxonomy is questioned based on the present work. Carrizo García et al. (2016) showed that *C. galapagoense* is nested among the closely related *C. frutescens, C. chinense,* and *C. annuum* Linaeus 1753 as demonstrated also by Ince et al. (2010). In a more recent study, Shiragaki et al. (2020) indicated that the *C. chinense* clade might be divided into two groups. Similarly, Tripodi et al. (2021) demonstrated that *C. chinense* species grouped in two clusters.

**Figure 1.**
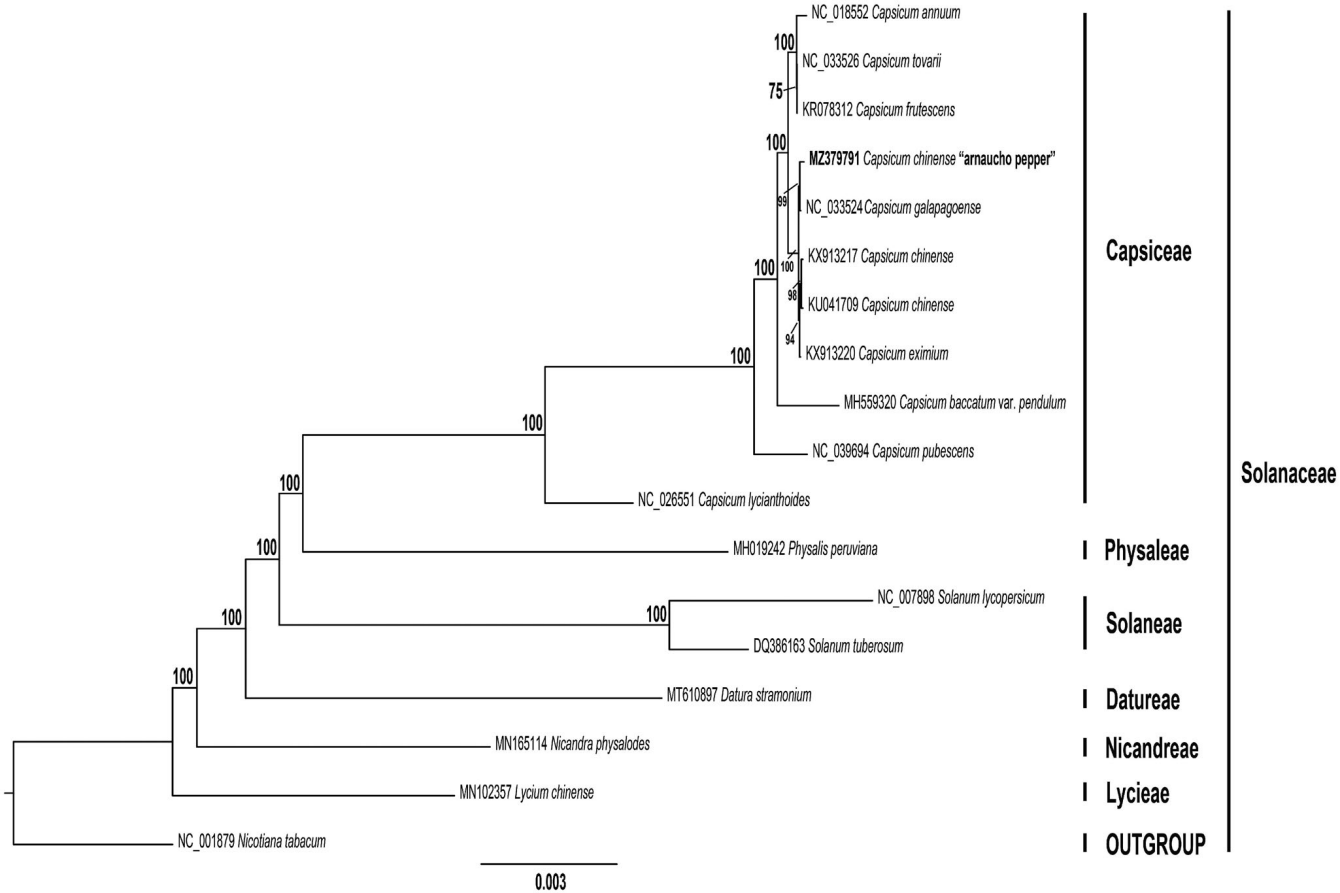
Maximum likelihood reconstruction of the 18 (including arnacucho chili pepper, *C. chinense*) whole chloroplast genome sequences, and one outgroup. Numbers above the branches represent bootstrap values, with only values higher than 70% shown. Names given to clades refer to the tribes in Solanaceae. Branch length (number of substitutions per site) is represented by a scale bar (bottom).

To our best knowledge, this is the first report of a cp genome of a Peruvian chili pepper landrace. However, we consider that further studies using additional collections of *Capsicum* from a wider geographic area and examination of relevant type material are needed to provide a better understanding of taxonomic variation and nomenclature in the *Capsicum* clade. A similar process was followed by Arbizu, Ellison, et al. (2016); Arbizu, Simon, et al. (2016) and Martínez-Flores et al. (2016) solving the species boundaries in another problematical group in *Daucus*. Our next step is to continue developing additional molecular tools for the arnaucho chili pepper that may shed light on elucidating its evolutionary history and promoting its adequate sustainable management, conservation and modern breeding.

## Ethical approval

Research and collection of plant material was conducted according to the guidelines provided by INIA. Permission was granted by grower of “arnaucho” chili pepper to carry out research on this landrace.

## Data Availability

The genome sequence data that supports this study is openly available in Genbank of NCBI under the accession number MZ379791 (https://www.ncbi.nlm.nih.gov/nuccore/MZ379791.1/). The associated Bioproject, Biosample and SRA numbers are PRJNA739476, SAMN19789523, and SRR14868519, respectively.
